# Composite Oncocytoma and Papillary Renal Cell Carcinoma of the Kidney Treated by Partial Nephrectomy: A Case Report

**DOI:** 10.1100/tsw.2011.120

**Published:** 2011-06-09

**Authors:** Michael S. Floyd, Saqib Javed, Keloth E. Pradeep, Alan R. De Bolla

**Affiliations:** ^1^ Department of Urology, Wrexham Maelor Hospital, Wrexham, UK; ^2^ Department of Pathology, Wrexham Maelor Hospital, Wrexham, UK

**Keywords:** renal, oncocytoma, papillary, partial

## Abstract

We present the case of a 73-year-old woman who presented with lethargy and a nonproductive cough. Computerised tomography of her abdomen revealed a 38-mm mass in the lower pole of her left kidney. She underwent a partial nephrectomy, with final histopathological analysis confirming the presence of a concomitant oncocytoma and papillary cell carcinoma. To our knowledge, this is the only case report in the world literature describing a papillary renal cell carcinoma within an oncocytoma treated by partial nephrectomy.

## INTRODUCTION

There are reports of oncocytomas existing in tandem with other tumours, such as phaechromocytomas and chromophobe carcinomas[1,2], and demonstrating unusual biological behaviour[3]. However, papillary tumours and oncocytomas have a different cell of origin and, hence, the presence of an oncocytoma and a concomitant papillary renal cell carcinoma is exceptionally rare[4]. We present such a case in a 73-year-old female who was diagnosed incidentally and treated with nephron-sparing surgery

## CASE REPORT

A 73-year-old female presented to the medical service with a 2-week history of lethargy, anorexia, and a nonproductive cough. Her past history was remarkable for cataract repair, hip replacement, and oesophageal reflux disease. There was no history of haematuria or abdominal pain. Physical examination was noncontributory. Initial laboratory investigations revealed a normal full blood count and renal profile. Liver function tests revealed a raised alkaline phosphatase of 168 IU/l and adjusted calcium of 2.89 µmol/l. C-reactive protein was elevated at 168 IU/l. A computerised tomogram of the abdomen was requested to investigate her gastrointestinal symptoms and hypercalcaemia. This revealed a 38-mm mass in the lower pole of her left kidney; staged as a T1 lesion suspicious for a renal cell carcinoma. She underwent a left partial nephrectomy and recovered uneventfully.

A partial nephrectomy specimen measuring 45 × 43 × 33 mm was received. Its cut surface showed a circumscribed, yellowish-brown tumour measuring 36 mm in maximum dimension and situated 8 mm from the resection margin. Microscopically, the tumour showed a nesting, alveolar, and tubular pattern, with uniform round/polygonal cells with abundant, intensely eosinophilic, and granular cytoplasm. The cells contained uniform, small, round, central nuclei with evenly dispersed chromatin without prominent nucleoli (Fig. 1). No mitoses were detected. Immunohistochemically, these cells were positive for E-cadherin and CD117, and negative for CK20, CK7, CD10, AMACR (Alpha Methyl CoA Racemase), and vimentin (Fig. 2). The appearances were those of an oncocytoma. Within this tumour, at one end, a separate 1.5-mm mass was noted. This second, well-demarcated tumour exhibited papillary architecture containing cells with prominent nucleoli and clear, eosinophilic, granular cytoplasm. Psammoma bodies were present (Fig. 1). Exfoliated, vacuolated cells were present in the lumina. Immunohistochemically, the tumour cells were negative for E-cadherin and CD117, and positive for CK7, CD10, and AMACR (Fig. 2). The exfoliated cells were positive for vimentin. The appearances were those of a type 2, papillary renal cell carcinoma, Furhman nuclear grade 2.

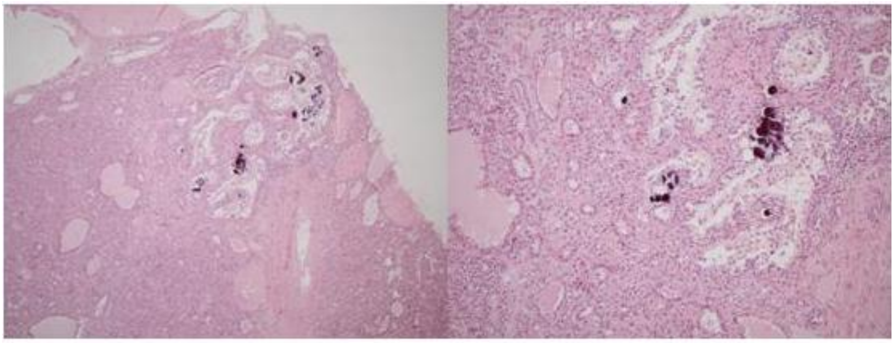

**FIGURE 1**. Haematoxylin & eosin staining showing a well-demarcated papillary renal cell carcinoma containing psammoma bodies within an oncocytoma.

## DISCUSSION

A 1976 review of renal cell carcinomas identified the first series of 13 oncocytomas[5]. As a neoplastic entity, they exhibit considerable biological variation, but are thought to originate from the intercalated cells of the collecting duct. Specific criteria aid in their histological diagnosis[6]. Macroscopically, the tumours should be cortically located, homogenous, mahogany brown, and contain a central scar without necrosis. Microscopically, cells should be polygonal with absent mitotic activity and an eosinophil-rich cytoplasm. Immunohistochemically, oncocytomas exhibit positivity for CD117 (c-kit) and E-cadherin, and negativity for CK7, AMACR, and CD10. Certain chromosomal alterations have been demonstrated: loss of chromosome 1 and the Y chromosome. Cytogenetically, translocations in the 11q13 region and trisomy, loss of heterozygosity, and monosomy are found.

Papillary renal cell carcinomas account for 10–15 % of all renal cell carcinomas and two subtypes exist. Type 1 tumours consist of papillae with a single layer of cells with scanty cytoplasm and low-grade nuclei. Type 2 papillary tumours contain pseudostratified cells that cover the eosinophilic cytoplasm and contain higher-grade nuclei. Chromosomal aberrations include loss of the Y chromosome, in addition to chromosomes 7 and 17. Certain heritable traits, such as mutations in the tyrosine kinase domain of the MET proto-oncogene, are responsible for hereditary papillary renal cell carcinoma[7]. The histogenesis remains ill defined, but the cell of origin is thought to be the proximal or distal convoluted tubule. Immunohistochemically, this tumour is positive for CK7, AMACR, and CD10, and usually negative for CD117 (c-kit) and E-cadherin.

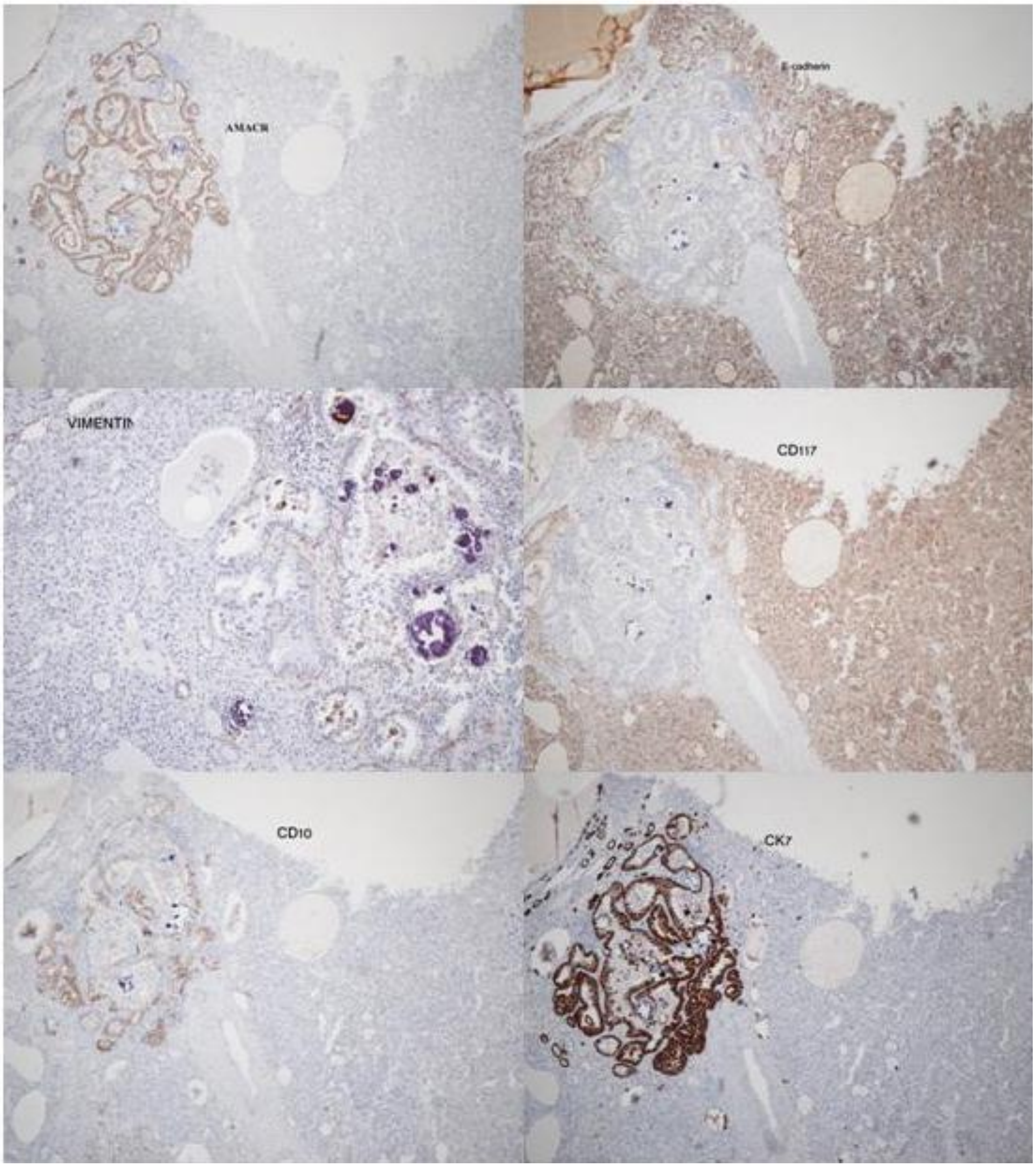

**FIGURE 2.** Immunohistochemical staining showing an oncocytoma positive for E-cadherin and CD117, but negative for CK7, CD10, and AMACR. The reverse was found in the papillary carcinoma. The exfoliated cells stained positive for vimentin.

Familial conditions, such as the Birt–Hogg–Dube syndrome, allow for the concomitant existence of oncocytomas with other renal cell tumours that exhibit common cytological features, such as chromophobe carcinomas[8]. Radiological confirmation remains a challenge as tomography is frequently unable to differentiate between oncocytomas and malignant renal neoplasms[9].

Some reports have described the coexistence of a nonfunctioning phaechromocytoma and an oncocytoma[1]. There are case reports of renal oncocytomas that have metastasised with presumed bony secondaries[3]. Other authors have described renal oncocytomas that have developed liver metastases almost a decade after nephrectomy[10]. Okada et al. described an oncocytic papillary carcinoma in an 81-

year-old woman[11]. This was shown to stain positive for AMACR (indicating papillary renal cell carcinoma), but negative for CD117. It is accepted that renal oncocytoma can coexist with chromophobe carcinoma in a pathological spectrum due to their common cell of origin[2], with oncocytoma accounting for the benign element and chromophobe the malignant phase. Papillary tumours and oncocytomas have different cells of origin, and in our case, two separate tumours were detected. The second tumour existed within the oncocytoma, but demonstrated separate, distinct features of papillary architecture with psammoma bodies; essentially a tumour within a tumour. In this case, the oncocytoma stained positive for E-cadherin and CD117, and negative for CK7, AMACR, and CD10, and the reverse was true for the papillary carcinoma. Immunohistochemical features of clear cell, papillary, and chromophobe renal cell carcinoma and oncocytoma are tabulated in Table 1.

**TABLE 1**
**Immunohistochemical Features**

| Tumour Type | CK7 | CK20 | AMACR | CD117 | CD10 | E-Cadherin | Vimentin |
|---|---|---|---|---|---|---|---|
| Clear | -ve | -ve | -ve | -ve | +ve | -ve | +ve |
| Papillary | +ve | +ve | +ve | -ve | +ve | +ve/-ve | +ve |
| Oncocytoma | -ve | +ve/-ve | -ve | +ve | -ve | +ve | -ve |
| Chromophobe | +ve | -ve | -ve | +ve | -ve | +ve | -ve |

## CONCLUSION

Burger at al. previously described a case of dual oncocytomas occurring in a single kidney, treated with nephron-sparing surgery, 4 years after a total nephrectomy for a papillary neoplasm in the contralateral kidney[12]. The dual pathological finding of an oncocytoma and papillary renal tumour in a single kidney is rare and we believe this to be the first case reported in the world literature treated by partial nephrectomy.
